# Supplementary material for: Expression of Fibrosis-Related Genes in Liver and Kidney Fibrosis in Comparison to Inflammatory Bowel Diseases
Source: Cells. 2022 Jan 18;11(3):314. doi: 10.3390/cells11030314 (PMC8834113; doi:10.3390/cells11030314)
Supplement: Supplementary file 1 [file cells-11-00314-s001.zip › cells-1518269-supplementary.pdf]

Table S1. Bioinformatics analysis of gene expression between fibrotic and normal tissue in kidney and liver. logFC: logarithm of fold change.

	**Kidney**		Liver	
**hgnc_symbol**	**logFC**	*p*-Value	logFC	*p*-Value
LYZ	3.166496	3.07×10^{-6}	1.476008	5.75×10^{-6}
COL1A1	3.102875	1.45×10^{-5}	2.351975	2.51×10^{-16}
PTPRC	2.956456	1.30×10^{-5}	1.282094	5.78×10^{-8}
CTSS	2.633633	3.65×10^{-6}	1.068561	3.80×10^{-9}
COL1A2	2.607892	7.80×10^{-5}	2.6464	1.58×10^{-17}
CXCL9	2.540713	0.000406	1.947122	2.75×10^{-8}
CD52	2.530327	3.39×10^{-6}	1.329524	4.58×10^{-9}
IGSF6	2.454723	9.30×10^{-7}	1.040033	5.41×10^{-8}
COL3A1	2.396587	4.97×10^{-5}	1.88969	1.36×10^{-15}
HLA-DQA1	2.192921	7.60×10^{-7}	1.898518	0.007945
HLA-DQA2	2.192921	7.60×10^{-7}	1.016686	1.05×10^{-5}
HLA-DQB1	2.139251	6.65×10^{-5}	1.52969	4.91×10^{-10}
COL14A1	1.949228	0.000551	1.430269	1.49×10^{-11}
IGHG1	1.930691	0.002785	1.715181	2.23×10^{-6}
IGHG2	1.930691	0.002785	1.715181	2.23×10^{-6}
IGHGP	1.930691	0.002785	1.715181	2.23×10^{-6}
GZMA	1.893884	2.29×10^{-5}	1.127769	2.48×10^{-8}
IGLC2	1.858849	0.003838	1.625392	3.17×10^{-7}
IGLC3	1.858849	0.003838	1.625392	3.17×10^{-7}
IL7R	1.831663	3.85×10^{-6}	1.103621	3.91×10^{-8}
CCL19	1.766256	0.000423	1.721957	4.61×10^{-7}
LUM	1.707301	2.89×10^{-6}	2.251182	1.02×10^{-12}
DCN	1.64195	7.75×10^{-5}	1.111262	2.58×10^{-13}
CCL5	1.61021	1.57×10^{-5}	1.115946	4.02×10^{-8}
APOBEC3G	1.589117	4.33×10^{-6}	1.105895	1.19×10^{-11}
TRBC1	1.538034	2.66×10^{-5}	1.320928	3.06×10^{-10}
TRBC2	1.538034	2.66×10^{-5}	1.320928	3.06×10^{-10}
TRIM22	1.530449	7.73×10^{-5}	1.244158	4.00×10^{-13}
STAT1	1.51646	0.000184	1.439039	1.85×10^{-10}
GABBR1	1.506491	0.000161	2.574328	1.27×10^{-10}

UBD	1.506491	0.000161	2.574328	1.27×10^{-10}
THBS2	1.473771	4.57×10^{-5}	1.898496	1.58×10^{-15}
IFI16	1.457906	9.81×10^{-6}	1.014602	2.83×10^{-11}
GBP1	1.451519	0.004152	1.099306	4.78×10^{-8}
SRPX	1.447139	0.000542	1.03087	5.73×10^{-10}
CLDN11	1.430348	0.000788	2.617619	8.67×10^{-18}
RNASE6	1.378641	4.48×10^{-5}	1.044119	1.11×10^{-10}
IGHA1	1.338536	0.010808	1.281697	3.03×10^{-6}
IGHA2	1.338536	0.010808	1.281697	3.03×10^{-6}
DPT	1.334792	0.00232	1.260204	4.09×10^{-8}
TNFAIP8	1.320408	1.28×10^{-6}	1.1053	2.54×10^{-10}
CELF2	1.307684	1.28×10^{-6}	1.033407	5.69×10^{-10}
HLA-DMA	1.292565	1.93×10^{-6}	1.172066	1.23×10^{-10}
IGKV1-39	1.261016	0.024654	1.638739	5.98×10^{-7}
IGKV1D-39	1.261016	0.024654	1.638739	5.98×10^{-7}
AKR1B10	1.216679	0.010233	3.518628	1.05×10^{-10}
IGKC	1.10921	0.007786	1.371007	1.15×10^{-7}
ANXA2	1.082011	5.26×10^{-7}	1.392319	1.07×10^{-12}
FRMD6	1.025143	0.000573	1.057359	5.96×10^{-10}
MGP	1.008158	0.009309	1.889504	8.65×10^{-12}
DKK3	1.005058	0.002548	1.943372	4.44×10^{-18}
NAP1L1	-1.0709	2.61×10^{-5}	1.69522	1.48×10^{-9}
PHLDA1	-1.0709	2.61×10^{-5}	1.69522	1.48×10^{-9}
RETREG1	-1.08024	0.000311	-1.04874	1.85×10^{-9}
CCN1	-1.56367	3.19×10^{-9}	1.424701	6.28×10^{-10}
LINC01554	-1.79924	2.06×10^{-7}	-1.94096	2.10×10^{-8}
PDK4	-2.52341	9.45×10^{-6}	-1.35882	6.68×10^{-7}

Table S2. Pearson's correlation coeficients among genes expressions within each samples group. Normal liver/normal kidney/liver fibrosis/kidney fibrosis. *$p < 0.05$; **$p < 0.01$.

JN/LN/JF/LF	THBS2	MGP	PTPRC	CD52	GZMA	DPT	DCN
CXCL9	-/0.715*/-/-	-/-/-/-	-/0.678*/0.524*/0.781**	-/0.738*/-/0.679*	-/-/-/0.831**	-/-0.749**/0.746**	-/-/-/-
THBS2		-/0.688*/-/0.774*	-/0.786*/0.663*/0.675*	0.862*/0.801**/0.490*/0.725*	-/-/-/-	-/-/-/-	-/-/0.582**/-
MGP			-/0.957**/-/0.789**	-/0.933**/-/0.646*	-/-/-/-	-/-/0.894*/0.685**	-/-/-/-
PTPRC				-/0.950**/0.610**/0.866**	0.977*/-/-/0.877*	-/-/-/-	-/-/-/-
CD52					0.949*/-/-/0.680*	-/-/-/-	-/-/0.557**/0.717*
GZMA						-/-/-/-	-/-/-/-
DPT							-/-/-/-

Table S3. Pearson's correlation coeficients among genes expressions within each samples group. Normal bowel/Ulcerative colitis/Crohn's disease without fibrosis/ Crohn's disease with fibrosis. *$p < 0.05$; **$p < 0.01$.

NC/UC/CDCV/CDC	THBS2	MGP	PTPRC	CD52	GZMA	DPT	DCN
CXCL9	-/-/-/-	-/-/-/-	-/-/0.638*/-	-/-/0.550*/-	-/-/-/0.727**	-0.634*/-/-/-	-0.641*/-/-/-
THBS2		0.857**/0.602*/-/-	-/-/-/-	-/-/-/-	-/-/-/-	0.690*/-/-/-	0.883**/0.834**/0.865**/0.844**
MGP			-/-/-/-	-/-/-/-	-/-/-/-	0.821**/0.729*/-/-	0.877**/-/0.577*/-
PTPRC				0.839**/0.829**/0.851**/0.806**	-/-/-/-	-/-/-/-	-/-/-/-
CD52					-/-/0.727*/-	-/-/-/-	-/-/0.556*/-
GZMA						-/-/-/-	-/-/0.738**/0.582*
DPT							0.867**/-/0.544*/-
